# A Randomized Trial of SMART Goal Enhanced Debriefing after Simulation to Promote Educational Actions

**DOI:** 10.5811/westjem.2017.11.36524

**Published:** 2017-12-21

**Authors:** Amish Aghera, Matt Emery, Richard Bounds, Colleen Bush, R. Brent Stansfield, Brian Gillett, Sally A. Santen

**Affiliations:** * Maimonides Medical Center, Department of Emergency Medicine, Brooklyn, New York; † Michigan State University College of Human Medicine, Spectrum Health Emergency Medicine Residency, Grand Rapids, Michigan; ‡ University of Vermont Medical Center, Division of Emergency Medicine, Department of Surgery, Burlington, Vermont; § Wayne State University School of Medicine, Detroit, Michigan; ¶ Virginia Commonwealth University School of Medicine, Richmond, Virginia

## Abstract

**Introduction:**

Goal setting is used in education to promote learning and performance. Debriefing after clinical scenario-based simulation is a well-established practice that provides learners a defined structure to review and improve performance. Our objective was to integrate formal learning goal generation, using the SMART framework (Specific, Measurable, Attainable, Realistic, and Time-bound), into standard debriefing processes (i.e., “SMART Goal Enhanced Debriefing”) and subsequently measure the impact on the development of learning goals and execution of educational actions.

**Methods:**

This was a prospective multicenter randomized controlled study of 80 emergency medicine residents at three academic hospitals comparing the effectiveness of SMART Goal Enhanced Debriefing to a standard debriefing. Residents were block randomized on a rolling basis following a simulation case. SMART Goal Enhanced Debriefing included five minutes of formal instruction on the development of SMART learning goals during the summary/application phase of the debrief. Outcome measures included the number of recalled learning goals, self-reported executed educational actions, and quality of each learning goal and educational action after a two-week follow-up period.

**Results:**

The mean number of reported learning goals was similar in the standard debriefing group (mean 2.05 goals, SD 1.13, *n*=37 residents), and in the SMART Goal Enhanced Debriefing group (mean 1.93, SD 0.96, *n*=43), with no difference in learning goal quality. Residents receiving SMART Goal Enhanced Debriefing completed more educational actions on average (Control group actions completed 0.97 (SD 0.87), SMART debrief group 1.44 (SD 1.03) *p*=0.03).

**Conclusion:**

The number and quality of learning goals reported by residents was not improved as a result of SMART Goal Enhanced Debriefing. Residents did, however, execute more educational actions, which is consistent with the overarching intent of any educational intervention.

## INTRODUCTION

In education, a critical step facilitating the transfer of lessons learned into practice is creating action plans or setting learning goals.^1^,^2^ While goals are not always accomplished, there is a clear relationship between setting goals and achievement.^3^,^4^ Goals can influence performance by focusing effort and attention to a specific domain resulting in greater effort and persistence of effort, as well as strategies to approach tasks.^3^–^5^ An established model for developing actionable learning goals is the “SMART” framework. These goals are Specific, Measurable, Attainable, Realistic, and Time-bound. The SMART framework is easy to teach, easy to remember, and has been employed successfully across multiple disciplines, including medical education.^6^–^12^ Ideally, SMART goals consist of practical, concrete actions that learners plan to implement to improve their knowledge, skills, and attitudes, with an emphasis on tangible outcomes.^7^,^9^,^13^

It is commonly held that residents will form learning goals without prompting and then execute them; however, this assumption is untested. While formal goal-setting instruction improves the quality of resident-generated learning goals, learners may struggle to independently create high-quality goals due to problems inherent in self-assessment.^14^–^17^ However, the practice of self-assessment has been shown to generate a greater number of learning goals, and these goals are more likely to be carried out.^8^,^18^

As an educational platform in healthcare, simulation-based medical education (SBME) lends itself as a strategy for pairing informed self-assessment and targeted goal setting. SBME employs well-structured, guided debriefing sessions incorporating formative feedback to impact performance.^19^–^23^ Debriefing strategies are designed to engage learners through a reflective conversation using objective feedback and self-assessment, thereby providing the context to change suboptimal practice patterns and improve patient outcomes.^24^ However, all debriefing techniques do not incorporate the generation of explicit learning goals.^25^ The use of debriefing in SBME as a vehicle to impact educational outcomes by providing informed self-assessment in conjunction with explicit goal-setting warrants further study.

The objective of our study was to compare the effectiveness of a novel debriefing modality that integrated the creation of quality, self-directed learning goals identified from a clinical simulation scenario, compared to a standard simulation debriefing without explicit dialogue about learning goals. We hypothesized that this “*SMART Goal Enhanced Debrief*” would result in the completion of a greater number and higher quality of learning goals and educational actions.

## METHODS

### Study Design

This was a prospective multicenter randomized controlled study comparing the effectiveness of a standard debriefing process to SMART Goal Enhanced Debriefing, which employed the use of coaching to develop “SMART” learning goals.^9^ Learners participated in a high-fidelity, mannequin-based clinical simulation scenario followed by formal debriefing with one of two methods. Measured outcomes included both the generation of learning goals and the subsequent completion of educational actions. The study was approved by each institution’s local institutional review board and classified as exempt at each site (i.e., informed consent was not required in accordance with standard educational practices).

**Population Health Research Capsule**

What do we already know about this issue?

Goals help to promote learning and performance. The “SMART” (Specific, Measurable, Attainable, Realistic, Time-bound) framework for setting goals has been successfully used across multiple disciplines including medicine.

What was the research question?

To evaluate the effectiveness of a SMART Goal Enhanced Debriefing strategy after simulation.

What was the major finding of the study?

SMART Goal Enhanced Debriefing stimulated additional self-directed learning through executed educational actions.

How does this improve population health?

Improving debriefing methodology after simulation has the potential to reach a wide variety of learners across the healthcare continuum.

### Study Setting and Sample

The study was conducted at three academic hospitals from November 2013 to March 2014, each supporting Accreditation Council for Graduate Medical Education approved residencies in emergency medicine (EM). Attributes include one Midwest urban university affiliated site with an annual emergency department (ED) census of 110K visits (Site1); one Mid-Atlantic suburban university affiliated site with an ED volume of 115K visits (Site 2); and one Northeast private urban site with an annual ED census of 120K visits (Site 3). Respectively, each site supports nine EM residents/year, 12 EM or combined program EM/family practice or EM/internal medicine residents/year, and 16 EM residents/year. Subjects included a convenience sample of EM residents or combined program residents. Participation in the study was voluntary, though residents were required at their respective institutions to routinely participate in simulation-based educational activities as part of general curricular requirements.

We determined necessary sample size based on estimated number of educational actions that would be reported in the control and intervention groups, based on the study team’s previous experience in this area.^8^ Initially, the need for 88 residents was predicted based on an estimate of 0.8 reported actions in the control group, and 2.0 in the intervention group (standard deviation [SD] 2, alpha 0.05, power 80%, enrollment ratio 1). We terminated enrollment early due to achieving statistical significance between the two groups.

### Study Protocol

#### Simulation Case Scenarios

**Figure 1:**
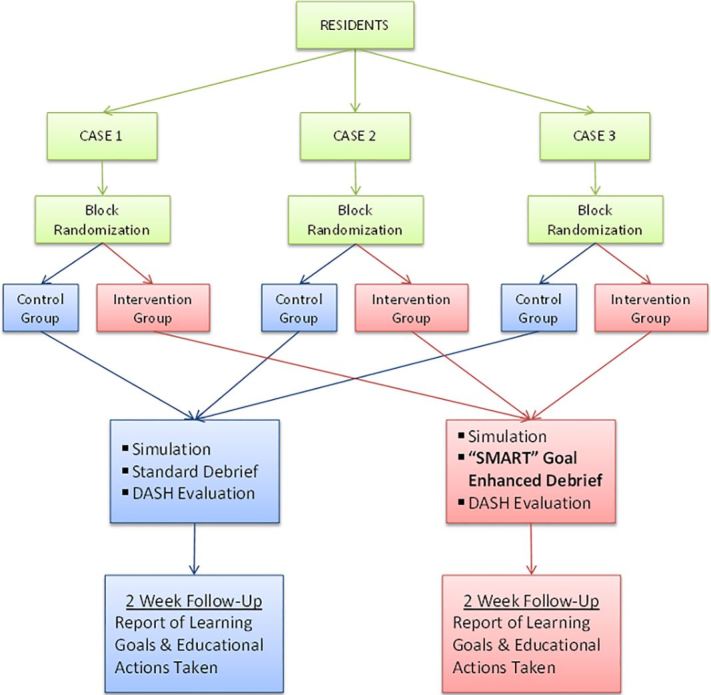
Schematic of study protocol comparing development of learning goals.

A schematic of the study protocol is graphically represented in Figure 1. Case scenarios were not standardized across institutions in order to model typical educational settings representing the variety of cases used for teaching. Recognizing that certain types of cases may lend themselves better as a stimulus for generating goals and actions, residents were block randomized by case at each site. The priority of randomization was to have a similar spread of cases across both groups. Program administrators did appropriately match resident postgraduate year (PGY) level to specific case scenarios and associated learning objectives in advance. Cases at each site involved the participation of two or three residents. Residents were enrolled only once and were blinded to their assigned group.

#### Debriefing

After completing the simulation, residents received approximately 30 minutes of debriefing time structured as a standard debrief (control group), or a SMART Goal Enhanced Debrief, which embedded five minutes of formal instruction and development of SMART learning goals (intervention group). Of note, the length of time for case scenarios and debriefing were constrained by each site’s curricular structure, and thus any individual group did not receive any more or less instruction time in total. Residents were asked to keep scenario details confidential to allow cases to remain novel for future participants.

EM academic faculty members with experience in simulation debriefing facilitated the simulation sessions. Faculty members were not limited to members of the study team or participation in either the control or intervention groups. However, to minimize the effect of varying debriefing styles each facilitator was trained to assure that each debriefing session was conducted in a well-accepted and structured format consisting of three phases: reactions, analysis/reflection, and summary/application (Appendix 1).^24^ It is important to note that facilitators would still routinely discuss lessons learned and next steps in the summary/application phase of the debrief as part of standard practice in the control group. The enhancement of this practice in the intervention group specifically related to coaching and writing down goals in the SMART format during this final debriefing phase.

#### SMART Goal Enhanced Debriefing

In the intervention group, education around the development of SMART learning goals was conducted in the summary/application phase of the debriefing to facilitate linking lessons learned from the case to explicit goals. Faculty instructors guided residents to generate SMART learning goals in response to the simulation, using a standardized worksheet that defined SMART learning goals with examples (Appendix 2). Residents were allowed to keep the worksheet after the debriefing.

#### Evaluation of Debriefing

At the conclusion of each debriefing session, residents were asked to complete the Debriefing Assessment for Simulation in Healthcare (DASH) for the purpose of monitoring the overall quality of SBME sessions in both study groups. Residents were given the “DASH – Student Version Short Form,” which is designed for learners to rate their instructors in each of the six core DASH elements in less than three minutes.^26^ Content validity of the DASH has its basis in best debriefing practices defined by an expert panel grounded in an extensive literature review.^27^

### Measurements

The primary outcome was to compare the number and quality of learning goals and educational actions recalled after a two-week follow-up interval by residents after standard debriefing (control group) to the learning goals and educational actions recalled by resident’s who underwent SMART Goal Enhanced Debriefing (intervention group). Specifically, all residents were asked to list learning goals and educational actions taken in response to their simulation case encounter (Appendix 3). A two-week time interval was chosen because the study team felt that it would be unlikely for educational actions to be executed beyond that time frame. Additionally, minimizing the follow-up period would help limit recall bias.

#### Learning Goal Rating Scale – Validity Evidence

**Figure 2:**
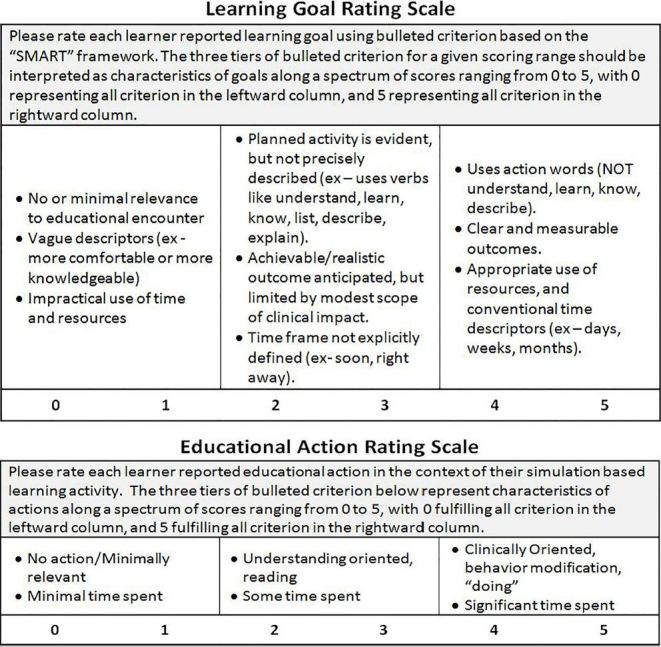
Learning goal and educational action rating instruments.

Initially, we rated the quality of learning goals using a scoring rubric with validity evidence published by Lockspeiser,^28^ which was subdivided into domains based in the “I-SMART” mnemonic (i.e., important, specific, measureable, etc.). Unfortunately, raters in this study could not reliably apply Lockspeiser’s rubric to the recalled goals submitted by our cohort of learners. As a result, we created a modified Learning Goal Rating Scale (Figure 2). To support content validity, we adapted Lockspeiser’s original anchors that uniquely related to the “SMART” criteria within the context of our learning-goal worksheet. Response process was improved through an iterative process of rater training and tool refinement. Developing general scoring guidelines and streamlining the tool into a single global rating scale decreased variation in interpreting the anchors.

Internal structure of the Learning Goal Rating Scale was supported by measuring an intraclass correlation coefficient (ICC), using a two-way model estimating the reliability of average κ ratings. Upon finalizing the structure of the Learning Goal Rating Scale, four members of the study team used it to independently rate a representative subset of learning goals (*n*=21) with good reliability (ICC=0.82). Once this initial reliability was established, the same four members of the study team applied the Learning Goal Rating Scale to every reported learning goal (*n*=155). We found that good reliability was maintained (ICC=0.78). The Learning Goal Rating Scale was not tested for relationships to other variables or consequences.

#### Educational Action Rating Scale – Validity Evidence

We measured the quality of the educational actions using an Educational Action Rating Scale (Figure 2). It was developed de novo as there was no existing instrument for this purpose. To support content validity, we chose rating criteria based on principles of education pedagogy such as the cognitive domain of Bloom’s Taxonomy.^29^,^30^ In essence, higher ratings would be given to activities that incorporated active learning and were deemed more relevant to clinical practice. Furthermore, given that the amount of time spent engaged in a learning activity correlates with educational impact, duration of the activity would also result in an improved rating. To support response process validity, the instrument was piloted and revised using an iterative process to simplify the interpretation of specific rating criteria. Initially, four members of the study team rated a representative subset of educational actions from our cohort (*n*=18) with good ICC (0.86). At three months, excellent test-retest reliability was demonstrated on the same subset of educational actions (ICC=0.94). Follow-up ratings of every educational action (*n*=95) by the same four raters revealed good ICC (0.90). The Educational Action Rating Scale was not tested for relationships to other variables or consequences.

#### Average Quality Ratings

Learning goal and educational action ratings were performed by four study investigators blinded to study site and group (control or intervention). Each study investigator rated the quality of reported goals and actions for all study subjects. We created the Average Learning Goal Quality by averaging ratings of learning goals within each study group. The Average Educational Action Quality was calculated in a similar manner.

### Data Analysis

We evaluated sampling distribution of simulation cases using a chi-squared test, or Fisher’s exact test when the case frequency was <5 in any group. A *p* value of <0.05 was considered significant. We used descriptive statistics to summarize the number and quality of goals and educational actions. The number and quality of learning goals and educational actions from the control and intervention groups were compared using a t-test. We summarized DASH results with descriptive statistics and applied t-tests to determine statistically significant differences in the delivery of SBME sessions between groups. A *p* < 0.05 level was considered significant.

## RESULTS

A total of 80 residents were enrolled in the study: 37 in the standard debriefing (control) group, and 43 in the SMART Goal Enhanced Debriefing (intervention) group. A breakdown of the PGY level of study subjects in each group and site are detailed in Tables 1 and 2. Table 3 lists simulation case scenarios, their frequency of utilization, and a statistical measure of randomization.

Residents in the standard debriefing group (*n*=37) recalled a total of 76 learning goals and subsequently reported 36 educational actions performed. Residents in the SMART Goal Enhanced Debriefing group (*n*=41) recalled 79 goals and reported 59 actions performed. Two PGY1 residents in the SMART Goal Enhanced Debriefing group were lost to follow-up at Site 3 (did not return/submit their learning goals and action items).

The mean number and quality of learning goals recalled and educational actions reported are detailed in Table 4. There was no significant difference in the mean number of goals reported or goal quality; however, residents receiving SMART Goal Enhanced Debriefing completed more educational actions on average (*p*=0.03). There was no difference in action quality.

We reviewed the DASH ratings of the simulation sessions in both groups to ensure that the quality of debriefing was similar in both groups. Both were rated similarly across all measured domains (Table 5).

## DISCUSSION

The ability to efficiently engage in goal-oriented, self-directed learning has the potential to serve as a scaffold for ongoing performance improvement over the entirety of a physician’s career. Widespread application of deliberate goal setting should be considered an important skill to promote ongoing professional development. In this study, SBME motivated residents to set learning goals after both standard debriefing as well as SMART Goal Enhanced Debriefing. Residents did not generate more learning goals as a result of receiving SMART Goal Enhanced Debriefing. Notably, residents from this group reported performing more educational actions, which is arguably the more important metric related to improving one’s clinical performance.

We theorize that the *process* of creating SMART learning goals served as a subconscious primer for the *execution* of goals. Priming is thought to improve the likelihood of one’s acting on a goal by increasing motivation, focus, and commitment.^3^,^31^ Concurrently, automatic goal activation can be influenced by associations with situational features and mental representations of colleagues’ goal pursuits.^32^ Both of these factors likely came into play in our study. For example, a key situational feature was the explicit use of SMART learning-goal worksheets, while debriefing with peers and instructors provided external mental representations of the goals of others.

Other educational factors may also have worked in combination, or even synergistically, to promote the execution of goals. For example, all simulation debriefings in our study used the technique of summarizing lessons learned in relation to observed performance. When explicitly linked with the development of learning goals, this technique may have served as a powerful stimulus to promote the completion of subsequent learning activities.^5^ Further codifying learning goals into the structured SMART framework may also have stimulated ongoing motivation such that even more actions were completed in the intervention group. Theoretical constructs in goal-setting supporting motivation include improving affect (i.e., feels good to achieve a goal); metacognition (i.e., stimulation of task strategies for goal attainment); and choice (i.e., learner-centered goals are more likely to be pursued).^4^,^5^

Regardless of the underlying mechanism, we believe that equipping learners with an *explicit* method to develop focused learning goals may help them become self-directed learners. This is particularly valuable in the context of SBME, which is a commonly employed educational technique across the healthcare continuum. Regardless of profession, simulation educators craft clinical cases and debriefing objectives tailored to their learners. Debriefing incorporates self-assessment and reflection as key components that impact the learning process. Building on this framework, improving a learners’ ability to create actionable learning goals will ultimately facilitate improvement in subsequent clinical performance. In our experience, instructors can become skilled at applying the SMART goal format in a short time period.

## LIMITATIONS

There are several limitations to this study. We chose to study our intervention with non-standardized simulation case scenarios to replicate conditions in routine educational settings in the hopes of making our findings more generalizable. While we asked all residents to self-report their learning goals and actions approximately two weeks after the educational encounter, it is difficult to know if residents accurately represented these goals and actions in follow-up. There may be an effect of recall bias. Finally, novel measurement tools were developed in an effort to quantify the quality of goals and actions. We recognize that our interpretations cannot be “fully valid.”^33^ As a result, validity evidence was collected during the development of the measurement tools. This resulted in a process of refinement of a Learning Goal Rating Scale. Similarly, development of the Educational Action Rating Scale was developed de novo and has not been validated externally. The impact on study results are unknown.

## CONCLUSION

We found that debriefing after simulation is an effective modality to stimulate the development of learning goals and the execution of educational actions. While the application of a simple goal-setting exercise (i.e., SMART Goal Enhanced Debriefing) did not increase the number and quality of goals recalled, it did serve as a powerful primer to promote additional self-directed learning through *executed* educational actions. This intervention can be readily applied to most simulation debriefing sessions and requires little training to be employed effectively.

## Supplementary Information







## Figures and Tables

**Table 1:** Subjects in the standard debriefing group.

Residents	Site 1	Site 2	Site 3	Total
PGY1	3	3	6	12
PGY2	5	3	4	12
PGY3	4	3	4	11
PGY4	0	2	0	2
PGY5	0	0	0	0

*PGY*, post graduate year.

**Table 2:** Subjects in the SMART Goal Enhanced Debriefing group.

Residents	Site 1	Site 2	Site 3	Total
PGY1	6	5	7	18
PGY2	4	5	5	14
PGY3	3	2	4	9
PGY4	0	0	0	0
PGY5	0	2	0	2

*PGY*, post graduate year; *SMART*, specific, measurable, attainable, realistic and time-bound.

**Table 3:** Clinical simulation case scenarios and frequency.

Simulation case scenario title	Standard debriefing frequency (*n*)	Goal enhanced debriefing frequency (*n*)	*p* value
Torsades	12	13	0.90
Bradycardia	6	6	0.82
Neuroleptic malignant syndrome	2	2	0.64
Unstable tachycardia	2	4	0.40
Hepatic encephalopathy	1	2	0.55
TCA overdose	1	1	0.72
Methanol toxicity	1	2	0.55
Cognitive error – right ventricular infarct	3	2	0.44
Placental abruption	2	6	0.18
Symptomatic bradycardia calcium channel blocker overdose	1	1	0.72
Penetrating neck trauma	3	0	0.10
Penetrating chest trauma	3	0	0.10
Carbon monoxide toxicity	0	2	0.30
Traumatic subarachnoid hemorrhage	0	2	0.30

*TCA*, tricyclic antidepressant.

**Table 4:** Mean and standard deviation of number and quality of learning goals and educational actions.

	Number of learning goals*	Average learning goal quality†	Number of educational actions*	Average educational action quality†
Standard debriefing (*n* = 37)	2.05 (1.13)	2.84 (0.88)	0.97 (0.87)	2.88 (0.89)
SMART-goal enhanced debriefing (*n* = 41)	1.93 (0.96)	2.88 (0.81)	1.44 (1.03)	3.01 (0.91)
*p* value	0.59	0.76	0.03	0.52

*SD*, standard deviation; *SMART*, specific, measurable, attainable, realistic, time-bound.

* mean per resident/SD

† mean/SD

**Table 5:** Mean and standard deviation of resident “DASH” ratings (Debriefing Assessment in Healthcare). Ratings are all reported on a scale of 1 to 7 (1=extremely ineffective, 7=extremely effective).

Individual DASH elements	Standard debriefing (*n* = 30)	SMART-goal enhanced debriefing (*n* = 35)	*p* value
Introduction to the simulation environment	5.9 (1.0)	6.1 (0.8)	0.34
Engaging context for learning	6.5 (0.7)	6.3 (0.8)	0.16
Organized debriefing structure	6.5 (0.6)	6.5 (0.7)	1
Provoked reflection of performance	6.4 (0.7)	6.5 (0.6)	0.34
Identified what was done well and poorly	6.0 (0.8)	6.2 (0.7)	0.44
Helped determine how to improve or sustain good performance	6.5 (0.6)	6.4 (0.7)	0.50
