# Supplementary material for: A Randomized Trial of SMART Goal Enhanced Debriefing after Simulation to Promote Educational Actions
Source: West J Emerg Med. 2017 Dec 21;19(1):112–20. doi: 10.5811/westjem.2017.11.36524 (PMC5785177; doi:10.5811/westjem.2017.11.36524)
Supplement: Supplementary file 1 [file wjem-19-112-s001.docx]

**Appendix 1:** Facilitator Debriefing Guidelines

"Debriefing" learners after a simulated clinical scenario allows for the methodical review of what happened and why.  Engaging participants in a logical stepwise approach while simultaneously fostering self-reflection results in deeper learning, and can ultimately de-bias error prone behaviors. In doing so, facilitators should provide a safe learning environment where participants can freely explore their thought processes and actions. Generally speaking, the role of the facilitator is to guide the discussion rather than to lecture at the participants.

*STEP 1:  "Blow off steam"*
Allow learners to express their initial reactions to the SIM.  These comments provide an important framework on which to build feedback in the context of their self-assessments.  One effective strategy is to simply ask "how did it go", and allowing the learner to answer uninterrupted. Pitfalls include defensive reactions to their comments about the case or ridiculing performance damage the underlying psyche of the learner.

*STEP 2:  Analyze performance*
Allow learners to make sense of the SIM, address their concerns, and then guide them through a discussion of the case objectives.  Eliciting the thought process of the learner is critical, so as to be able to work with them in the clinical context to rethink actions so that performance will improve in the future.  Furthermore, generalizing the clinical context to a broad array of situations is a powerful method to enact behavior change on a larger scale compared to an individual scenario.  While taking the necessary time to correct negative behaviors is paramount, it is also important to provide feedback which reinforces positive behaviors. Other pitfalls include telling learners what to do without self-reflection, or running through teaching points without tying into specifics of the clinical scenario.

*STEP 3:  Summarize*
Translate lessons learned into memorable principals that will stick with the trainees.  Simply asking "what have you learned" is sufficient.  If learners don't cover an important point, summarizing it for them will still help to reinforce the teaching point.  Pitfalls include ending a debrief abruptly, monopolizing the discussion, or not stressing the connections to clinical practice.
