# Supplementary material for: A Randomized Trial of SMART Goal Enhanced Debriefing after Simulation to Promote Educational Actions
Source: West J Emerg Med. 2017 Dec 21;19(1):112–20. doi: 10.5811/westjem.2017.11.36524 (PMC5785177; doi:10.5811/westjem.2017.11.36524)
Supplement: Supplementary file 2 [file wjem-19-112-s002.docx]

**Appendix 2:** Learning Goal Worksheet

The generation of learning goals is a valuable method for helping learners to improve performance and achieve competence. Prior research has demonstrated that learning goals are most effective when they are “SMART”, as defined below with examples.

- ***S****pecific -* detailed and focused; describe exactly what is to be accomplished
- ***M****easurable -* possible to determine whether you have achieved the goal
- ***A****chievable* - can you do it? Is it possible?
- ***R****ealistic -* feasibility; can you incorporate the goal into your schedule?
- ***T****ime bound -* include a specified time frame

| **Specific** | *Not specific:* “I want to improve my ultrasound skills”  *Specific:* “I will do a supervised bedside ultrasound on the next 5 pregnant patients that I care for in order to improve my skill level” |
| --- | --- |
| **Measurable** | *Not measurable:* “I’ll try to do a better job of discussing code status”  *Measurable:* “I’ll have an attending observe my next family discussion of code status, and request feedback.” |
| **Achievable** | *Not achievable*: “I’ll read all of Annals of Emergency Medicine every month ”  *Achievable:* “I’ll read 2-4 articles per month on important emergency medicine topics” |
| **Realistic** | *Not realistic:* “I will read the entire chapter on cardiac emergencies tonight in Tintanelli”  *Realistic:* “I will read the section on pulmonary edema tonight.” |
| **Time bound** | *Not time bound:* “I’ll achieve this goal soon.”  *Time bound:* “I’ll review the literature by the end of the week.” |

Within the “SMART” framework, learning goals should be phrased in an active format using *action verbs* describing something that you will be able to do once the goal is completed. An ideal plan to successfully achieve a goal *incorporates multiple different learning resources, activities, or strategies*.

| **Based on today’s simulation and debrief, consider “SMART” learning goals that you would like to achieve, and list them below:** |
| --- |
|  |
|  |
|  |
|  |
|  |
|  |
|  |
