# Supplementary material for: A Randomized Trial of SMART Goal Enhanced Debriefing after Simulation to Promote Educational Actions
Source: West J Emerg Med. 2017 Dec 21;19(1):112–20. doi: 10.5811/westjem.2017.11.36524 (PMC5785177; doi:10.5811/westjem.2017.11.36524)
Supplement: Supplementary file 3 [file wjem-19-112-s003.docx]

**Appendix 3:** Follow-Up Questionnaire

*The purpose of this survey is to improve the educational value of our simulation sessions. Please be honest in your responses.* ***It is ok to report that you didn’t create any goals or perform any actions****, and it will not have any refection on you as a resident whatsoever. The educational response of learners to simulation is actually unknown, so we will also collect your responses as part of a research project. Again,* ***please respond truthfully****. Your time and effort to complete this survey is appreciated.*

| **Please list learning goals that you generated as a result of your recent simulation session.** A learning goal is a statement of the vision of your intended learning and describes what you want to be able to do once the goal is completed. **(if none, please list “N/A”):** |
| --- |
|  |
|  |
|  |
|  |
|  |
|  |
|  |

| **Please list actions you have taken since the simulation session to improve your skills, knowledge, or performance. (if none, please list “N/A”):** | **Time spent on each action** |
| --- | --- |
|  |  |
|  |  |
|  |  |
|  |  |
|  |  |
|  |  |
|  |  |
